# Clinical Utility of Serum CA 19-9 Level in Colorectal Carcinoma Patients With Normal Serum Carcinoembryonic Antigen Levels: Correlation With Clinicopathological Features and Survival Outcomes

**DOI:** 10.7759/cureus.113104

**Published:** 2026-07-21

**Authors:** Avisikta Mallick, Arkaprovo Roy, Ria Roy

**Affiliations:** 1 Surgical Oncology, All India Institute of Medical Sciences, Jodhpur, Jodhpur, IND; 2 General Surgery, North Bengal Medical College and Hospital, Kolkata, IND; 3 Community and Family Medicine, All India Institute of Medical Sciences, Patna, Patna, IND

**Keywords:** ca 19-9, colorectal cancer, early diagnosis, normal cea, prognosis, tumor markers

## Abstract

Introduction

Colorectal cancer (CRC) is a major global health concern and is the third most commonly diagnosed cancer worldwide, accounting for approximately 11% of all cancer cases. It is more common in men than women. While the incidence has been declining among older adults (>65 years), its incidence and case fatality rate have increased (2% and 1.3%, respectively) among those younger than 50 years. The American Society of Clinical Oncology recommends the measurement of carcinoembryonic antigen (CEA) for prognostication and postoperative follow-up in CRC. However, routine checking of the level of carbohydrate antigen 19-9 (CA 19-9) as a marker of colon cancer is not recommended. The problem lies in young patients and poorly differentiated histological varieties where CEA is usually not elevated, especially in earlier stages. Among other existing tumor markers, CA 19-9 is a well-studied marker that is elevated in many abdominal malignancies, especially of hepatobiliary origin. Here, we explored its role in CRC.

Objectives

Our primary objective was to determine the association between serum CA 19-9 and CRC in patients with a normal CEA value. The secondary objective was to determine the association of serum CA 19-9 level with various clinicopathological features and its impact on short-term survival.

Materials and methods

This retrospective-prospective observational study was conducted among the patients of the Department of General Surgery, Department of Surgical Oncology, and Department of Surgical Gastroenterology of Medical College and Hospital, Kolkata, West Bengal, India. Statistical analysis was carried out using Microsoft Excel 2019 (Microsoft Corporation, Redmond, WA) and SPSS version 20.0 (IBM Corp., Armonk, NY).

Results

The study cohort comprised 41 patients with histologically confirmed colorectal carcinoma, including 27 males and 14 females. The age distribution was almost equal (20 vs 21). Twelve patients had stage 1 disease, 9 had stage 2, 14 had stage 3, and 6 had stage 4 disease. The median CEA of all these patients was 3.3, and the median CA 19-9 was 15.1. Elevated serum CA 19-9 levels were significantly associated with poorly differentiated CRC. The median CA 19-9 level increased from stage I (10.0 U/mL) to stage III (29.5 U/mL). Elevated CA 19-9 values were found more often in advanced T categories (T3-T4)’ however, the median CA 19-9 value neither correlated with the individual T, N, or M stages nor with the overall disease stages. While there was a trend towards improved survival in node-negative patients, a statistically significant survival advantage was observed in patients with non-metastatic disease. There was also a slight survival advantage in well to moderately differentiated tumors over poorly differentiated varieties (17.27 months vs 15.73 months, respectively), though it was not statistically significant. The overall survival at 18 months follow-up did not change significantly across CA 19-9 levels.

Conclusion

In colorectal carcinoma patients with normal serum CEA levels, elevated serum CA 19-9 was associated with adverse clinicopathological characteristics, but it does not significantly affect short-term survival. These findings suggest that CA 19-9 may provide complementary clinical information in this selected patient population; however, larger prospective studies with multivariable analyses are needed to clarify its independent prognostic value.

## Introduction

Colorectal cancer (CRC) ranks as the third most common cancer worldwide and the fourth most diagnosed in India. It is also the second leading cause of cancer-related deaths globally as per the GLOBOCAN 2020 data [1]. Although it is a disease of the elderly population, recent data showed a steep rise of early onset CRC before age 50 [2,3]. Previously, CRC happened to be the disease of developed countries, now its incidence is increasing in developing countries as well due to a shift in lifestyle and food habits [4]. Therefore, early diagnosis is crucial for improving survival rates, yet a significant proportion of cases are diagnosed at advanced stages, especially in resource-poor countries like India. Screening programs offer a survival advantage, but challenges remain, including limited sensitivity, cost-effectiveness, invasiveness, and compliance.

Tumor markers have long been investigated for early cancer detection and prognosis. Carcinoembryonic antigen (CEA) was the first identified marker in colon cancer cells by Freedman and Gold and was eventually found in various other epithelial cells in the stomach, tongue, esophagus, cervix, and prostate. Also, it has been found that CEA level does not rise until a very advanced disease stage [5]. Therefore, its limited specificity necessitates the exploration of other markers. Carbohydrate antigen 19-9 (CA 19-9) is a carbohydrate moiety important in cell adhesion and helps in tumor progression. This was found to be elevated in various gastrointestinal malignancies [6]. However, routine CA 19-9 testing for CRC is not generally recommended. In this study, we aimed to identify any available biomarkers, e.g., CA 19-9, for early disease detection. Our primary objective was to determine the association of serum CA 19-9 levels with colorectal carcinoma when serum CEA is normal. The secondary objective was to determine its association with various clinicopathological features and its impact on short-term survival among CRC patients with normal CEA levels.

This article was selected in the e-poster category at the National Comprehensive Cancer Network (NCCN) Annual Congress 2022, and the abstract was published in their journal (JNCCN), volume 20, issue 3.5, on 31 March, 2022 [7].

## Materials and methods

This institution-based retrospective-prospective observational study was conducted after obtaining approval from the Institutional Ethics Committee, between March 2020 and June 2021, and was registered in the Clinical Trials Registry-India (CTRI/2021/09/036543).

This study included newly diagnosed CRC cases presenting to the Departments of General Surgery, Surgical Gastroenterology, and Surgical Oncology of Medical College and Hospital, Kolkata, West Bengal, India.

Inclusion criteria were as follows: (1) newly diagnosed, biopsy-proven colorectal adenocarcinoma, (2) normal preoperative serum CEA level, and (3) availability of complete clinical, pathological, and follow-up data.

Exclusion criteria included the following: (1) elevated preoperative serum CEA levels, (2) receipt of any prior treatment for CRC, including surgery, chemotherapy, or radiotherapy, (3) history of another primary malignancy, and (4) benign conditions known to elevate serum CA 19-9 levels, such as pancreatobiliary disease, cholangitis, pancreatitis, or other inflammatory gastrointestinal disorders.

Patient data, including medical history, symptoms, clinical examination findings, and laboratory investigations, were collected. Serum samples were obtained before definitive surgery or neoadjuvant/palliative treatment. Surgical specimens were assessed by the Department of Pathology, Medical College and Hospital, Kolkata, and reviewed at the Oncopathology laboratory before adjuvant treatment. Serum CEA and CA 19-9 levels were determined using a standard chemiluminescent microparticle immunoassay. The cut-off values were 2.5 ng/mL for non-smokers and 5 ng/mL for smokers for CEA [8,9], and 37 U/mL for CA 19-9 [10].

A total of 41 patients fulfilled the eligibility criteria. Of these, four patients were identified retrospectively, while the remaining 37 patients were enrolled prospectively.

Statistical analyses were performed using Microsoft Excel 2019 (Microsoft Corporation, Redmond, WA) and SPSS version 20.0 (IBM Corp., Armonk, NY). Preoperative hemoglobin, bilirubin, and albumin levels were expressed as means. Serum CA 19-9 levels, due to non-normal distribution, were expressed as median and interquartile range. The Mann-Whitney U test was used to compare median CA 19-9 levels with different variables. Survival analyses were conducted using the Mantel-Cox log-rank test and Kaplan-Meier survival curves over a median period of 18 months.

## Results

The study cohort comprised 41 patients with histologically confirmed colorectal carcinoma, including 27 males and 14 females. The age distribution was nearly equal, with 20 patients aged ≤50 years and 21 patients >50 years. Baseline characteristics, including preoperative hemoglobin and albumin levels, were similar in patients with normal and elevated CA 19-9 levels. Cholestasis was not observed in any patient. Rectal and rectosigmoid growth was observed in approximately half of the patients (51.21%). Three patients presented with colonic perforation, 12 with obstruction, and 26 with neither. The majority of patients (60.97%) had T3-T4 lesions, and node positivity was almost uniform (48.78% node-positive vs. 51.21% node-negative). Of the total 41 patients, 35 (85.36%) patients had no evidence of metastasis, whereas six (14.63%) patients had biopsy-proven metastasis. Of these six patients, two patients had diffuse peritoneal disease, one had metastatic thyroid nodule, and the rest three had hepatic metastasis. Patients were also categorized according to their overall disease stage. Twelve patients had stage 1 disease, 9 had stage 2, 14 had stage 3, and 6 patients had stage 4 disease. The median CEA of all these patients was 3.3, and the median CA 19-9 was 15.1. Table 1 shows the various clinicopathological features of patients among groups with elevated CA 19-9 levels and normal CA 19-9 levels.

**Table 1:** Comparison of clinicopathological characteristics between patients with elevated and those with normal preoperative serum CA 19-9 levels Data are presented as n (%) unless otherwise specified. Statistical analysis: categorical variables were compared using Fisher's exact test. Continuous variables were compared using Student's t-test or Mann–Whitney U test, as appropriate. CA 19-9, carbohydrate antigen 19-9; CEA, carcinoembryonic antigen

Variable	Elevated CA 19-9 (n = 14)	Normal CA 19-9 (n = 27)	p-Value
Age, years			
≤50	5 (35.7)	15 (55.6)	1
>50	9 (64.3)	12 (44.4)	
Sex			
Male	7 (50.0)	20 (74.1)	0.494
Female	7 (50.0)	7 (25.9)	
Hemoglobin, mean (g/dL)	10.01	10.25	0.69
Bilirubin, mean (mg/dL)	0.5	0.43	-
Albumin, mean (g/dL)	3.29	3.53	0.3
Tumor location			
Right colon	3 (21.4)	7 (25.9)	-
Left colon	2 (14.3)	8 (29.6)	
Rectosigmoid/rectum	9 (64.3)	12 (44.4)	
T stage			
T1–T2	2 (14.3)	14 (51.9)	0.041
T3–T4	12 (85.7)	13 (48.1)	
N stage			
N0	7 (50.0)	14 (51.9)	0.186
N+	7 (50.0)	13 (48.1)	
Metastatic disease at presentation			
No	11 (78.6)	24 (88.9)	0.393
Yes	3 (21.4)	3 (11.1)	
Histological differentiation			
Well/moderately differentiated adenocarcinoma	6 (42.9)	22 (81.5)	0.022
Poorly differentiated/mucinous/Signet-ring adenocarcinoma	8 (57.1)	5 (18.5)	
CEA, median (ng/mL)	3.98	3.02	0.42
CA 19-9, median (U/mL)	75.01	4.15	-

**Figure 1:**
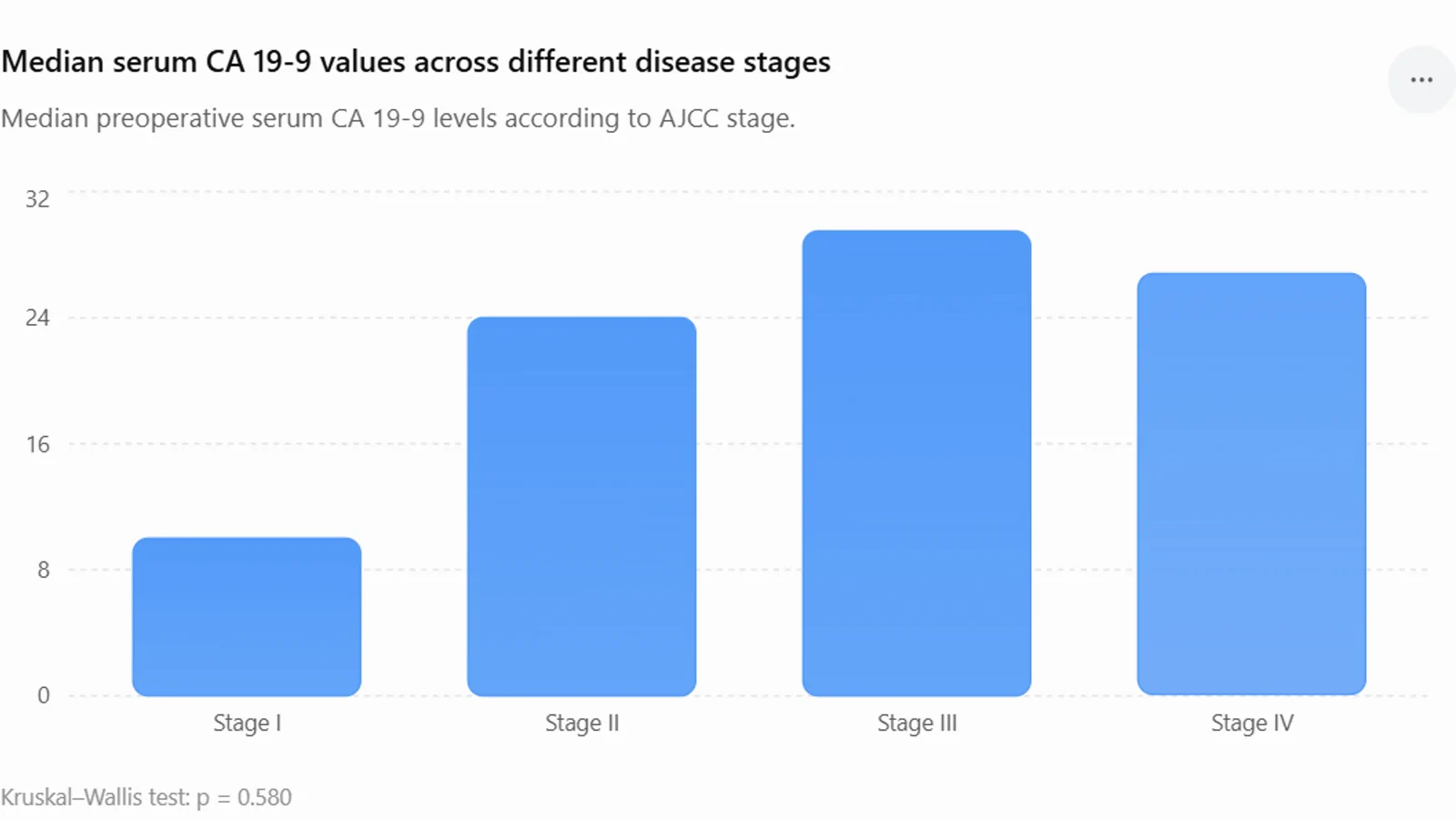
Distribution of median CA 19-9 values across disease stages (stage 1: 10 U/mL; stage II: 24 U/mL; stage III: 29.5 U/mL; stage IV: 26.8 U/mL) AJCC, American Joint Committee on Cancer; CA 19-9, carbohydrate antigen 19-9

There was no statistically significant difference in median serum CA 19-9 levels across the American Joint Committee on Cancer (AJCC) stages I-IV (Kruskal-Wallis H = 1.963, p = 0.580). The median serum CA 19-9 level tended to increase with advancing disease stage, with the highest median value observed in stage III disease (Figure 1). However, the overall difference in CA 19-9 levels across AJCC stages was not statistically significant (Kruskal-Wallis test, p = 0.580).

When patients were categorized according to serum CA 19-9 status, elevated CA 19-9 levels (>37 U/mL) were significantly more frequent among patients with advanced primary tumors (T3-T4) than among those with T1-T2 tumors (Fisher's exact test, p = 0.041). No significant association was observed between serum CA 19-9 levels and nodal status or metastatic disease (Table 2).

**Table 2:** Association between T stage and elevated serum CA 19-9 levels CA 19-9, carbohydrate antigen 19-9; CEA, carcinoembryonic antigen

T Stage	Normal CA 19-9 (n)	High CA 19-9 (n)	Fisher's exact test (p)
T1–T2 (n = 16)	14	2	0.041
T3–T4 (n = 25)	13	12	-

**Figure 2:**
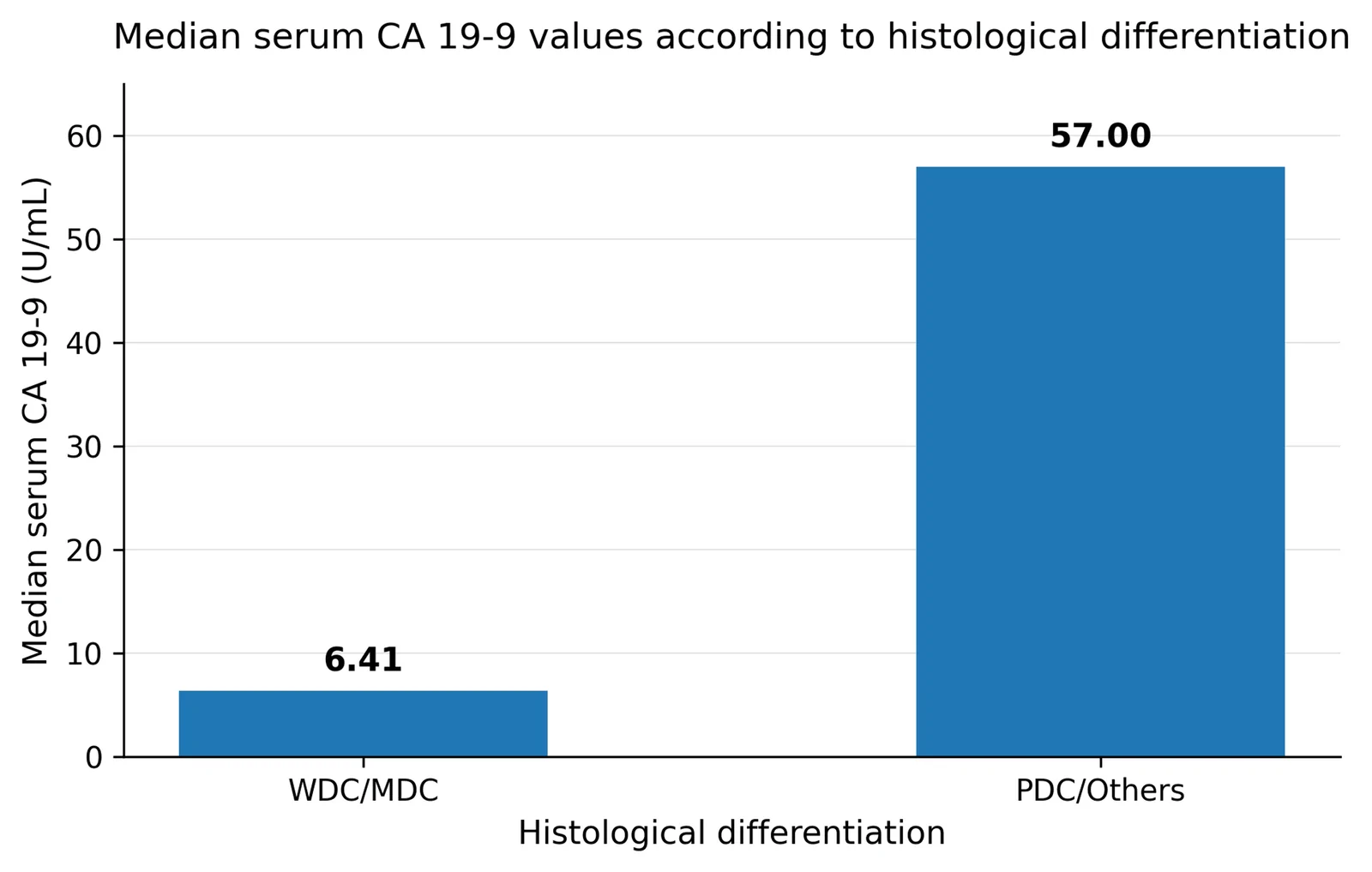
Distribution of median serum CA 19-9 values in different histological grades MDC, moderately differentiated carcinoma; PDC, poorly differentiated carcinoma; WDC, well differentiated carcinoma

Tumors were also categorized into well/moderately differentiated and poorly differentiated/other subtypes. Median serum CA 19-9 levels were significantly elevated in poorly differentiated and other unfavorable histological categories (median 57.0 vs 6.4 U/mL; Mann-Whitney U = 99.5, p = 0.022) (Table 3, Figure 2).

**Table 3:** Association between serum CA 19-9 with different histological categories CA 19-9, carbohydrate antigen 19-9; MDC, moderately differentiated carcinoma; PDC, poorly differentiated carcinoma; WDC, well differentiated carcinoma

Categories of histology	n	Median CA 19-9 (U/mL)	IQR	Mann–Whitney U	p-Value
WDC and MDC	28	6.41	25.44	99.5	0.022
PDC and others	13	57	48.7	-	-

Serum CA 19-9 levels did not differ significantly based on age, gender, disease presentation, or tumor location. There was no significant difference in mean survival time between age groups, gender, presentation groups, or T stages. A significantly higher CA 19-9 level was observed in patients who presented with intestinal obstruction (Table 4).

**Table 4:** Association of preoperative serum CA 19-9 level in the presence of obstruction CA 19-9, carbohydrate antigen 19-9

Categories of obstruction	n	CA 19-9 value	Mean rank	Mann-Whitney U	p-Value
		Median ± interquartile range			
Present	12	63.00 ± 174	28.21	87.5	0.013
Absent	29	7.00 ± 33	18.02		

**Figure 3:**
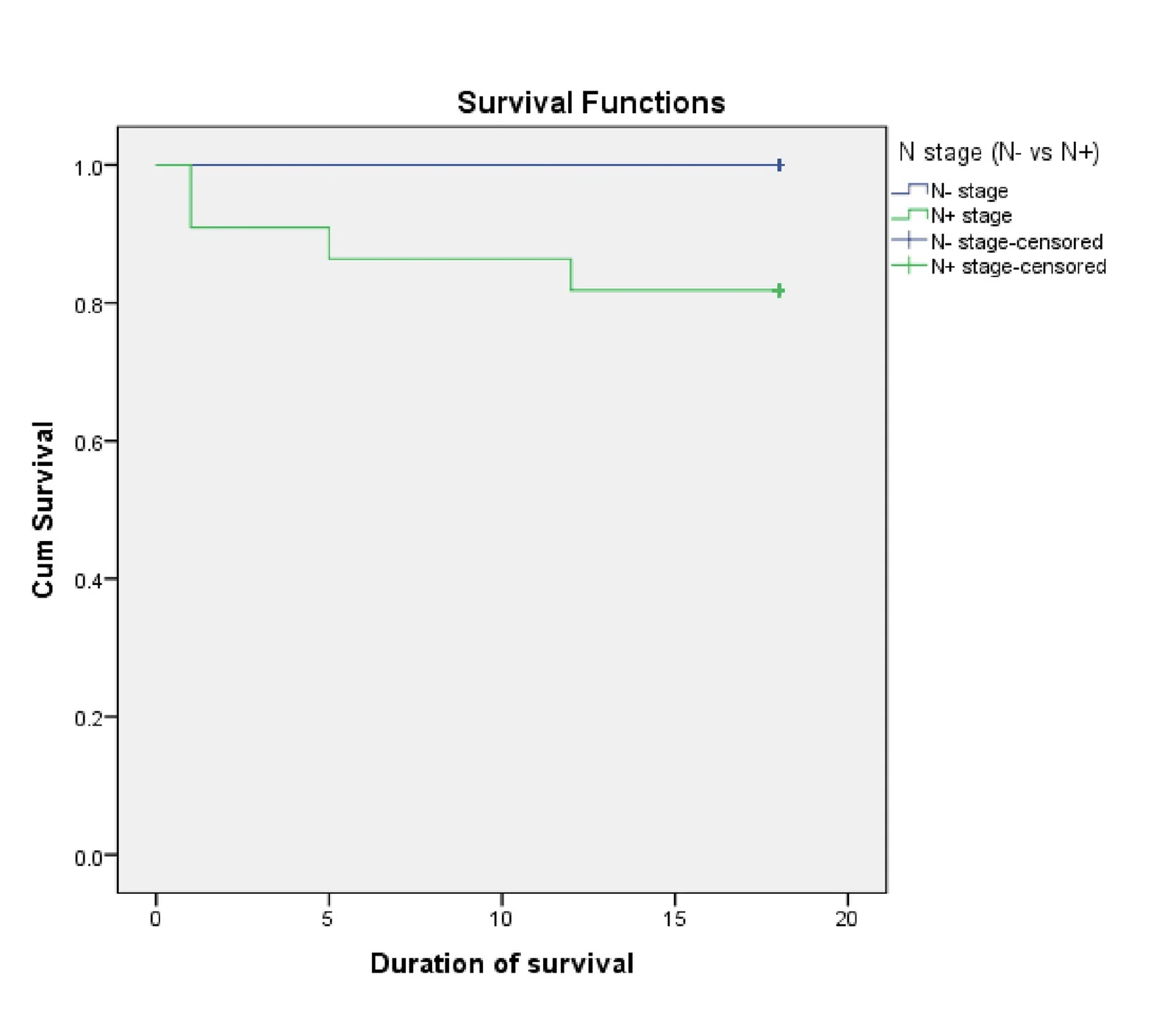
Kaplan-Meier curve on survival analysis as per the nodal stage The Mantel-Cox value is 0.054

**Figure 4:**
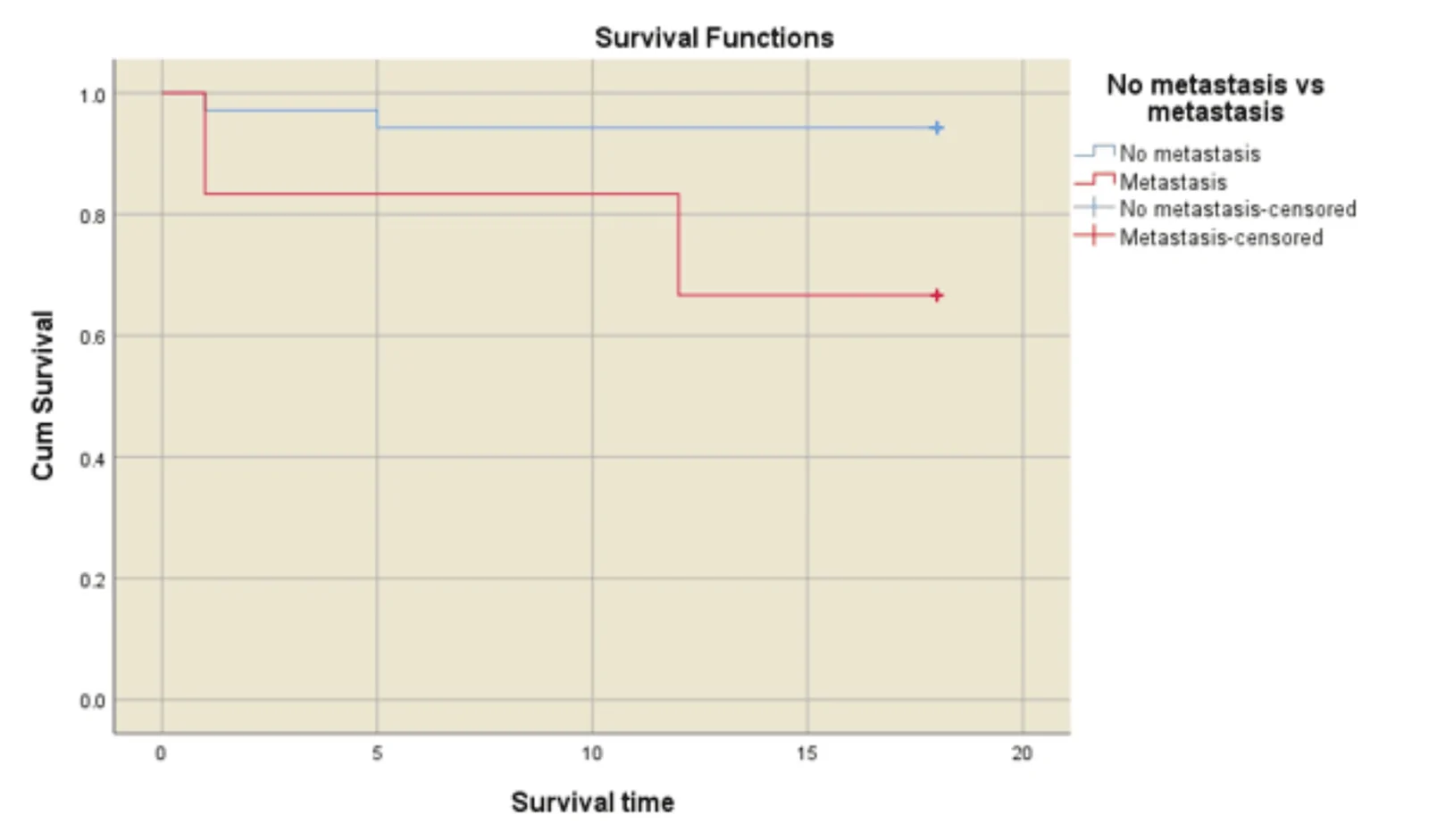
Kaplan-Meier survival analysis curve on the basis of presence of metastatic disease The p-value is 0.037 as per the Breslow (generalized Wilcoxon) score, and therefore it is statistically significant

After a median follow-up of 18 months, a controversial survival advantage was observed in node-negative disease (p=0.054), as well as a definite survival advantage in non-metastatic disease (p=0.037) (Figures 3, 4).

**Figure 5:**
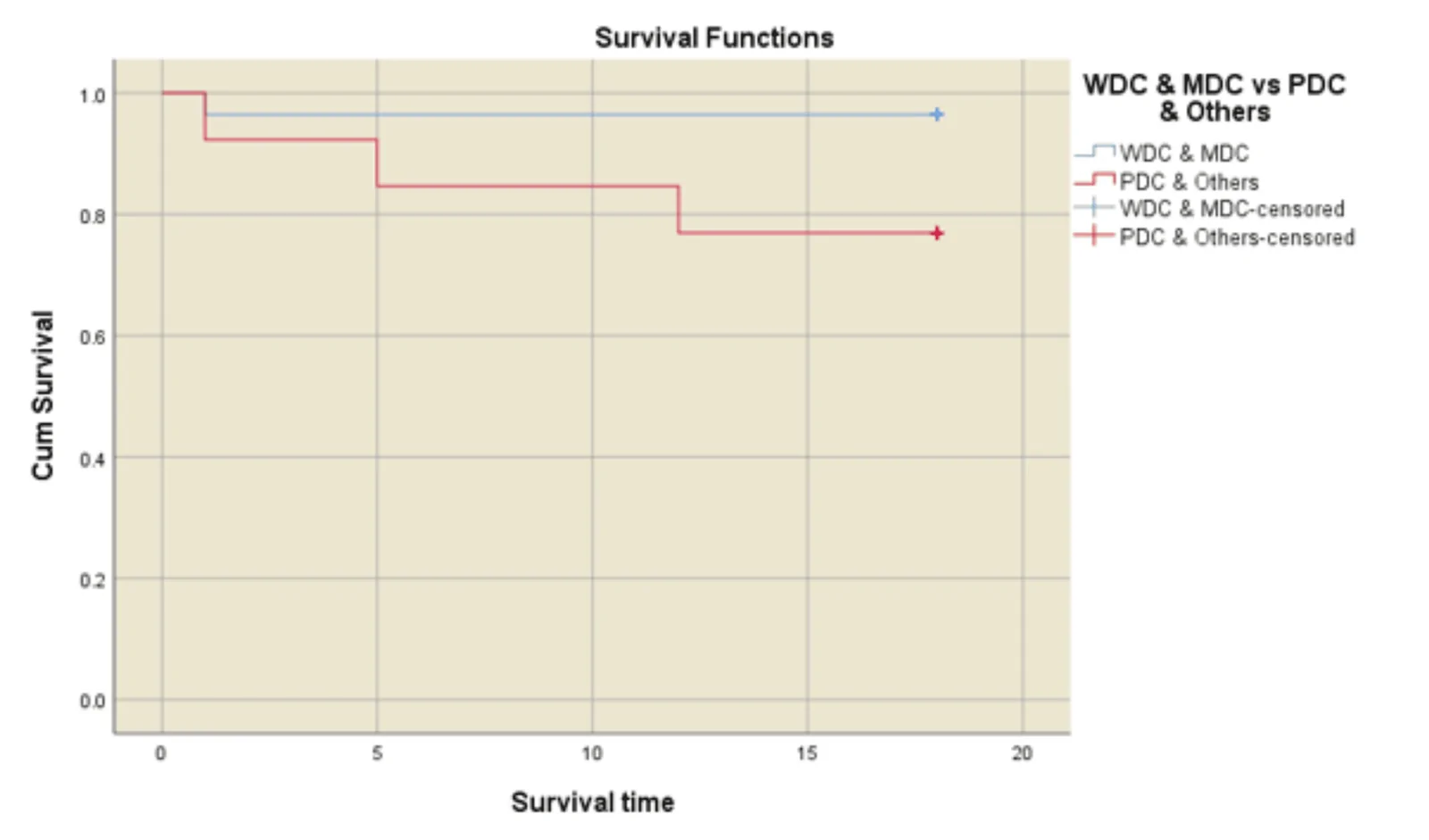
Kaplan-Meier survival analysis curve on the basis of histological categories Test of equality of survival distributions for the different histological categories (WDC & MDC vs PDC & Others such as signet ring cells and mucinous variety) has been done using the Breslow (generalized Wilcoxon) analysis. There is no statistically significant difference in survival time between two groups (p=0.059). MDC, moderately differentiated carcinoma; PDC, poorly differentiated carcinoma; WDC, well differentiated carcinoma

Patients with well- and moderately differentiated adenocarcinoma had a numerically longer mean overall survival than those with poorly differentiated carcinoma and other adverse histological variants; however, this difference did not reach statistical significance (Breslow test, p = 0.059) (Figure 5).

**Figure 6:**
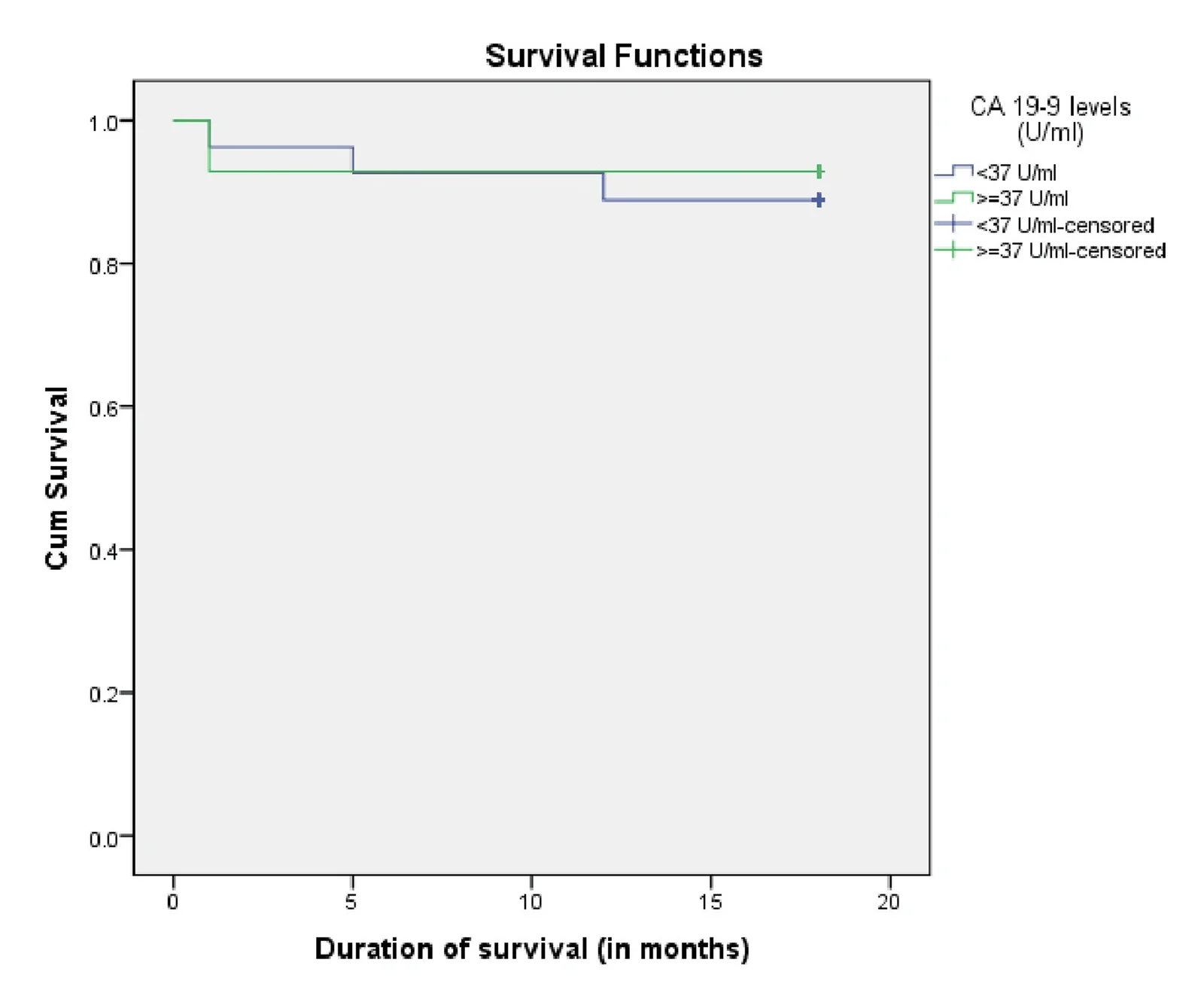
Kaplan-Meier survival analysis curve between patients having normal versus elevated CA 19-9 levels The Mantel-Cox log rank test value 0.47; hence, no significant difference in survival was stated as per preoperative CA 19-9 levels CA 19-9, carbohydrate antigen 19-9

Finally, no significant difference in survival was noted between groups having normal and elevated CA 19-9 levels (Figure 6).

## Discussion

CEA is not useful for detecting asymptomatic CRC, as its sensitivity and specificity are not high, particularly for early stages of disease. The role of CEA in CRC is mainly to monitor recurrence after curative treatment. Therefore, in this study, we included patients with normal CEA levels and explored the role of existing marker, i.e., CA 19-9 [11]. The diagnostic role of CA 19-9 as a test for the detection of colorectal malignancy remains poorly defined because, as in other diagnostic modalities, the utility of CA 19-9 has several confounding limitations such as false-positive elevations in many benign conditions. In 1984, Kuusela et al. studied a comparison between CA 19-9 and CEA in the serum of patients with a newly diagnosed, still untreated colorectal carcinoma and operated patients with residual tumor or a clinically confirmed relapse [12]. In their study, the sensitivity of CEA was 69% and that of CA 19-9 was 36%, while the combination of both markers achieved a sensitivity of 73%. The best specificity was obtained by the CA 19-9 estimation (97%), whereas the specificity was 70% using both tests. This suggested CA 19-9 as a useful additional marker in the diagnosis and follow-up of CRCs.

In the present study, we excluded patients who had cholestasis or other benign or malignant biliary diseases to remove the confounding factors. We also matched other baseline parameters (preoperative hemoglobin, albumin, etc.) in both groups (normal CA 19-9 and elevated CA 19-9).

We found that the median CA 19-9 level had no statistically significant difference among the different age and sex categories.

Nakatani et al. in their research from 2012 reported that the colon cancer located in the region of sigmoid had extremely high concentrations of CEA and CA 19-9 [13]. In our study, we did not find a significant difference in serum CA 19-9 levels between different tumor locations (right colon, left colon, and rectum and rectosigmoid lesions). This was similar to what was observed in the study carried out at Sarajevo University [14].

Here we also compared the serum CA 19-9 levels between the two aforementioned histological groups and found a statistically significant difference in the median CA 19-9 level (3.87 U/mL vs 42.7 U/mL; p=0.019), consistent with the Chinese study published in 2019 [15].

In the same study and another study carried out at Sarajevo University, Halilovic et al showed markedly elevated levels of CEA and CA 19-9 in colon cancers in stage IV disease compared to other stages. This elevation was more in high-risk groups than in low-risk groups [16]. In our study, there is a gradual rise in the median values of CA 19-9; however, the mean value was extremely high in stage IV due to the presence of an outlier. There was no statistically significant difference in median serum CA 19-9 levels across AJCC stages I-IV (Kruskal-Wallis H = 1.963, p = 0.580). Elevated CA 19-9 values were observed more frequently in advanced disease stages (T3-T4); however, no statistically significant difference was observed in median CA 19-9 values among individual T, N, and M stages. This can be explained by the fact that we did not stratify patients into high- and low-risk groups and that the number of metastatic diseases was also low in our study set.

The patients with obstructive pathology had significantly elevated CA 19-9 levels, but there was no significant difference between patients who had perforated lesions and those who did not. The significantly increased level of CA 19-9 can be explained by the fact that these tumors are mostly associated with advanced disease stage and aggressive tumor biology with mucinous differentiation, which are all associated with elevated CA 19-9 production.

Our study also evaluated the association of preoperative serum CA 19-9 level with survival after receiving curative treatment.

In the above study, the increased serum levels of these two biomarkers were associated with the histopathological size of the tumor. However, the linear regression model does not show any predictive significance of the stage or depth of invasion into the bowel wall [16]. Our study also failed to show any survival benefit in better T (p=0.098) and N stages (p=0.054). However, there was an improved overall survival noted among non-metastatic disease over the metastatic variety (p=0.035).

Although patients with well- and moderately differentiated histologies had a longer mean overall survival than those with poorly differentiated and other adverse histological variants (17.27 vs. 15.73 months), the difference was not statistically significant. This can be due to the small sample size and a very short follow-up period.

The Chinese study mentioned earlier also evaluated the prognostic value of pre-treatment serum CA 19-9 values. They showed that a high preoperative CA 19-9 level indicated a higher risk of lung and abdominopelvic metastasis and also poor prognosis in stage III patients. A multivariate analysis identified a high preoperative CA 19-9 level as an independent prognostic factor for three-year overall survival and disease-free survival [15]. They suggested incorporating serum CA 19-9 level into a standard TNM staging system to further refine risk stratification to predict benefits in overall survival from adjuvant chemotherapy, especially in stage III colon cancer. In the contrary, our current study did not show any survival advantage in patients with low preoperative serum CA 19-9 levels over high marker levels. This may be attributable to the limited sample size and the small proportion of patients with metastatic disease.

Finally, we can say that both of these tumor markers have got association with the advanced stages and poor histological varieties of the disease. However, their role in diagnosing the disease is still controversial. Therefore, there is a scope for a better understanding of the cellular and molecular pathogenesis of the disease, which can lead to the discovery of novel tumor markers to fulfil this gap.

The main limitation of our study was the poor sample size; in addition, the samples in each category was even smaller, making an accurate analysis difficult. The population was not categorized into high- and low-risk groups, which may have some impact on survival. The genetic study could not be done, which could have some correlation with the serum CA 19-9 level. The absence of multivariable survival analysis precludes assessment of CA 19-9 as an independent prognostic factor. Finally, the median follow-up period was too short to accurately comment on the overall survival.

## Conclusions

This study demonstrates that elevated serum CA 19-9 levels are associated with advanced primary tumor stage and adverse histopathological differentiation in patients with colorectal carcinoma and normal preoperative serum CEA levels. However, serum CA 19-9 was not associated with overall survival in this cohort; therefore, its independent prognostic value could not be established. These findings suggest that CA 19-9 may serve as a useful complementary biomarker for assessing disease burden in patients with normal CEA levels rather than as a standalone prognostic marker. Larger prospective studies with longer follow-up are needed to validate these findings and to determine whether the combined assessment of CA 19-9 and CEA provides additional prognostic value in colorectal carcinoma. Further research is also warranted to identify more sensitive biomarkers for the early detection of colorectal carcinoma, thereby facilitating timely diagnosis and increasing the potential for curative treatment.
